# Bacterial Biofilm and its Role in the Pathogenesis of Disease

**DOI:** 10.3390/antibiotics9020059

**Published:** 2020-02-03

**Authors:** Lene K. Vestby, Torstein Grønseth, Roger Simm, Live L. Nesse

**Affiliations:** 1 Department of Immunology and Virology, Norwegian Veterinary Institute, P.O. Box 750 Sentrum, N-0106 Oslo, Norway; lene.karine.vestby@vetinst.no; 2 Department of Otolaryngology, Head and Neck Surgery, Oslo University Hospital HF, Postboks 4950 Nydalen, 0424 Oslo, Norway; t.gronseth@gmail.com; 3 Institute of Oral Biology, University of Oslo, P.O. Box 1052, Blindern, 0316 Oslo, Norway; roger.simm@odont.uio.no; 4 Department of Food Safety and Animal Health Research, Norwegian Veterinary Institute, P.O. Box 750 Sentrum, N-0106 Oslo, Norway

**Keywords:** biofilm, otitis media, rhinosinusitis, endocarditis, wound infections, vaginosis, prostatitis, urinary tract infections, inflammatory bowel disease (IBD), cancer

## Abstract

Recognition of the fact that bacterial biofilm may play a role in the pathogenesis of disease has led to an increased focus on identifying diseases that may be biofilm-related. Biofilm infections are typically chronic in nature, as biofilm-residing bacteria can be resilient to both the immune system, antibiotics, and other treatments. This is a comprehensive review describing biofilm diseases in the auditory, the cardiovascular, the digestive, the integumentary, the reproductive, the respiratory, and the urinary system. In most cases reviewed, the biofilms were identified through various imaging technics, in addition to other study approaches. The current knowledge on how biofilm may contribute to the pathogenesis of disease indicates a number of different mechanisms. This spans from biofilm being a mere reservoir of pathogenic bacteria, to playing a more active role, e.g., by contributing to inflammation. Observations also indicate that biofilm does not exclusively occur extracellularly, but may also be formed inside living cells. Furthermore, the presence of biofilm may contribute to development of cancer. In conclusion, this review shows that biofilm is part of many, probably most chronic infections. This is important knowledge for development of effective treatment strategies for such infections.

## 1. Introduction

Bacteria form biofilms as part of their survival mechanisms, and biofilms are thus ubiquitous in nature. Already in 1683, Antoni van Leeuwenhoek observed and described biofilms by using his primitive microscope on matter from his own teeth. However, the biofilm lifestyle of microorganisms were of no interest to medical microbiologists until the early 1970s when Nils Høiby observed a link between the etiology of a persistent infection and aggregates of bacteria in cystic fibrosis patients [1]. Since then, biofilms have been recognized to be involved in many clinical infections [2,3], and evidence is accumulating that biofilms contribute to the pathogenesis, especially in chronic infections [4].

Bacterial biofilms are clusters of bacteria that are attached to a surface and/or to each other and embedded in a self-produced matrix. The biofilm matrix consists of substances like proteins (e.g., fibrin), polysaccharide (e.g., alginate), as well as eDNA. In addition to the protection offered by the matrix, bacteria in biofilms can employ several survival strategies to evade the host defense systems. By staying dormant and hidden from the immune system, they may cause local tissue damage and later cause an acute infection. Within the biofilm, the bacteria adapt to environmental anoxia and nutrient limitation by exhibiting an altered metabolism, gene expression, and protein production, which can lead to a lower metabolic rate and a reduced rate of cell division [3,5]. In addition, these adaptations make the bacteria more resistant to antimicrobial therapy by inactivating the antimicrobial targets or reducing the requirements for the cellular function that the antimicrobials interfere with. During a biofilm infection, simultaneous activation of both innate and acquired host immune responses may occur; neither of which are able to eliminate the biofilm pathogen, but instead accelerate collateral tissue damage [6]. Consequently, biofilm-related diseases are typically persistent infections that develop slowly, are rarely resolved by the immune system, and respond inconsistently to antimicrobial treatments.

In this paper, we have reviewed the scientific literature on a number of diseases in the various body systems suggested to be biofilm-related (Table 1), dental- and implant-related diseases not included. The main focus is placed on the scientific evidence for the presence of biofilm in these diseases and a possible role of biofilm in the pathogenesis. A growing amount of research deals with ways of combating such biofilms. However, this is not considered to be within the scope of this review.

## 2. The Auditory System

### Otitis Media

Otitis media (OM) is defined as an inflammation of the middle ear cavity. It is one of the most common causes of infection in pre-school aged children [7,8] and one of the most common causes of antibiotic prescription and surgical intervention in developed countries [9]. OM can be subdivided into acute OM (AOM), chronic supportive OM (CSOM), and OM with effusion (OME) [10]. These conditions can lead to temporary or permanent hearing loss [11].

Predisposing factors associated with the development of OM have been extensively studied. One is the anatomy of the Eustachian tube in infants and children <1 year of age. The tube has a shorter, wider, and more horizontal course that promotes the transmission of otopathogenic bacteria from the nasopharynx to the middle ear cavity, which increases the risk of OM [10]. Colonization of the nasopharynx by otopathogenic bacteria such as *Streptococcus pneumoniae*, and non-typeable *Haemophilus influenzae* (NT-Hi), in early childhood considerably increases the risk of subsequent episodes of AOM and OME later in life [12,13]. Although these findings pointed toward bacteria being important in the development of chronic otitis media, it was difficult to demonstrate residual bacterial colonization due to negative cultures in research studies in the past [14]. In spite of these negative cultures, studies were showing increasingly more evidences that bacteria were a part of the pathogenesis. Polymerase chain reaction (PCR) demonstrated bacterial DNA in the absence of positive cultures [15,16]. Reverse transcriptase-polymerase chain reaction (RT-PCR) showed metabolically active bacteria in the presence of negative culture in patients with OME. This led to the hypothesis that biofilms were a part of the pathogenesis of chronic otitis media and OME [17]. Later research found biofilms located in the middle ear of both humans and animal models, by a variety of different modalities like scanning electron microscopy and confocal laser scanning microscopy. Today, biofilms are generally considered a part of the pathogenesis of OM [18,19,20].

During the last decade, biofilms have been demonstrated in different subgroups of OM. In children with OME, bacterial aggregates indicating in vivo biofilms have been found in middle ear effusion. This confirms that biofilms can also be formed in the middle ear fluid and not only on the middle ear mucosa [21,22].

AOM is most common in children under 5 years of age [10]. Nearly 40% of the children have six or more episodes of AOM [9]. An association between AOM and biofilm in the nasopharynx (NP) has been suggested. Several studies have shown biofilms on the adenoids from children undergoing adenoidectomy for sleep apnea, recurrent and chronic OM [23,24]. It has been suggested that bacteria from the nasopharynx can detach and travel to the middle ear causing an acute infection [25]. This was highlighted by Kaur et al. who found the same sequence type of NT-Hi in 31 of 34 children from the NP and middle-ear fluid samples during an AOM [26].

Cholesteatoma is a condition where keratinizing squamous epithelium is trapped in the middle ear and/or in the mastoid process where biofilms have been demonstrated [19,27,28,29,30]. One main theory behind cholesteatoma formation is pneumatization failure and inflammatory conditions of the middle ear and mastoid cavity, leading to reduced middle-ear pressure. This can lead to retraction pocket formation, epidermal migration failure, and eventually cholesteatoma formation [31,32]. Since biofilms have been demonstrated in the middle ear and mastoid process mucosa, and the fact that biofilms have been linked to inflammatory changes in the mucosa and ventilation changes in the middle ear [33], one hypothesis is that a biofilm infection could cause favorable conditions for cholesteatoma pathogenesis and that cholesteatomas are favorable to biofilm [27]. Biofilms have been found in up to 81.3% of cholesteatomas [19,27,28,29,30] supporting the hypothesis that the keratinized matrix may be a favorable environment for biofilm formation. When a biofilm is established in a cholesteatoma it can lead to relapsing infections with draining ear and a recalcitrant course, which is commonly observed in aural cholesteatoma [27,32,34,35].

CSOM is a chronic inflammation of the middle ear and mastoid mucosa with a non-intact tympanic membrane from which discharge (otorrhea) is present [36]. It is one of the most common chronic infectious diseases worldwide, often occurring in the first 5 years of life and more common in developing countries [36,37]. There have been several scientific papers demonstrating biofilms in CSOM patients. These studies showed presence of biofilm in a range from 42% (in the mastoid mucosa) to 92% (when the middle ear is included) and being significantly more than in the controls [28,35,38]. Saunders on the other hand, found biofilm in only one out of seven patients [30]. In patients with recurrent OM (ROM), defined as three or more episodes in 6 months, biofilm was observed in 17 out of 20 children (21 out of 25 ears examined) in biopsies taken during undergoing tympanostomy tube placement [19]. Biofilms could be one reason for the relapsing nature for ROM and CSOM in spite of the appropriate antibiotic therapy. Orally administered antibiotics do not reach the concentrations in the middle ear that is needed to eradicate biofilms [39].

Tympanostomy tube placement is the most common surgical procedure in US children beyond the newborn period [40], and is used to treat chronic OME and RAOM with middle ear effusion [41]. Although the tube insertion are used in RAOM, it may be a cause of biofilm infection. Post-tympanostomy otorrhea is a common complication and in one meta-analysis occurred in 26% of patients (17% of intubated ears) during the intubation time [42]. One survey even found otorrea in 83% in an 18-month period [40]. There have been several studies to demonstrate biofilms on tympanostomy tubes [43,44]. Further to demonstrate the connection between biofilm and post-tympanostomy otorrhea, a study of persistent otorrhea 9 out of 15 patients showed positive labeling of extracellular DNA (eDNA) and integration host factor (IHF) in otorrhea samples indicating that biofilm may have a role in persistent otorrhea [45]. Failure of topical antibiotic therapy and persistent otorrhea may lead to the additional use of antibiotics and the need for tympanostomy tube removal [46,47].

## 3. The Cardiovascular System

### 3.1. Infective Endocarditis (IE)

The primary infection in endocarditis is a biofilm composed of both bacterial and host components located on the cardiac valve. This biofilm causes disease in the following ways: (1) The biofilm physically disrupts valve function, causing leakage when the valve is closed and turbulence as well as diminished flow when the valve is open; (2) the biofilm provides a source for near-continuous infection of the bloodstream that are difficult to remove by antibiotic treatment; (3) pieces of biofilm can break off and be carried to a terminal point in the circulation causing the brain, kidneys, and extremities particularly vulnerable to emboli.

It is generally accepted that biofilms is involved in infective endocarditis (IE) and it has been so for several years since Costerton et al. recognized native valve endocarditis as a biofilm infection caused by viridans group streptococci [2]. Treatment with antibiotics are often difficult even if the bacteria are sensitive to the selected antibiotic. Successful treatment with antibiotics often require prolonged intravenous administration. In cases where antibiotic treatment is unsuccessful, surgical excision and replacement of the infected valve might be an option. Most of what is known about the pathogenesis of endocarditis involving biofilms is learned from animal studies using rabbits [48]. The biofilm on the valve consists primarily of bacteria and biofilm matrix components, platelets and fibrin derived from the circulation. First the endothelial surface of the valve gets injured, second a formation of the sterile clot like lesion of platelets and fimbrin occurs at the site of the injury. Then bacteria starts to adhere to the thrombus before microcolonies are formed and lastly a mature biofilm is formed and pieces of the mature biofilm can cause embolization [48,49].

The most commonly isolated microorganisms from IE cases are staphylococci, streptococci, and enterococci. These species are responsible for more than 80% of IE cases. Electron microscopy is used for identification of biofilm in relation to endocarditis [49]. Already before Costerton et al. recognized endocarditis as a biofilm infection [2], Marrie et al. published a study in 1987 where bacterial colonies embedded in a matrix material on valves of six IE cases were shown using electron microscopy [50].

To diagnose IE, the patient is evaluated for several different criteria called Duke criteria. The criteria are based on clinical, echocardiographic, and microbiologic (blood culture) evaluation. However, bacteria of the biofilm rarely enter the blood stream as planktonic bacteria, and for this reason the blood culture may be negative when testing for microorganisms [51]. Consequently, immunodiagnostic assays (ELISA) have been developed to detect serum antibodies against biofilm matrix components. For example, an ELISA has been developed to detect antibodies against staphylococcal slime polysaccharide antigens. To date, the ELISA assays developed do not have the sensitivity and specificity to alone determine biofilm-associated infections [49].

### 3.2. Atherosclerosis

In atherosclerosis, fatty deposits and calcium accumulate as plaques in the arterial wall. This leads to reduced arterial elasticity, narrowed lumen of the artery, and subsequently to cardiovascular diseases caused by reduced blood flow. In addition, sudden rupture of a plaque may be life threatening [52]. A number of studies, including two recent meta-analyses, show that periodontal disease and cardiovascular disease, including atherosclerosis, are significantly related [53]. Furthermore, oral bacteria have been identified in the atherosclerotic plaques in several studies. However, it is not known whether the bacteria are involved in initiation of plaque formation or colonize the plaques after they are formed. Recently, bacterial biofilms in atherosclerotic arteries have been identified by fluorescence microscopy and fluorescence in situ hybridization (FISH) [54,55]. This may indicate biofilms to be involved in the pathogenesis of atherosclerosis, and it is hypothesized that the presence of biofilm may contribute to enhanced risk of plaque rupture [55].

## 4. The Digestive System

### 4.1. Sialolithiasis

Sialolithiasis is a condition where calcified masses (called sialoliths or salivary stones) form within a salivary gland. This can cause pain and swelling in about 0.5% of the general population. Earlier studies have suggested that bacteria may be involved in sialolithiasis [56], and new studies now link this to biofilm production. In a descriptive case-control study, sections of submandibular glands with chronic obstructive sialadenitis were compared with those of healthy controls, using confocal laser scanning microscopy [57]. Morphological evidence of bacterial biofilm was observed in half of the histological sections of the chronic obstructive sialadenitis group, whereas no sign of bacterial biofilm formation was seen in the control group. Interestingly, two recent studies report observations of biofilm structures in the center of the stones, thus indicating that biofilm formation may be part of the etiology of salivary stone production [58,59]. Fusconi et al. observed structures resembling bacterial cells embedded in amorphous material, when investigating the stones by scanning electron microscopy [59]. Furthermore, the presence of bacterial DNA was demonstrated by qPCR. In a later study, Kao et al. reported light and scanning electron microscopy observations of biofilm together with host immune cells, platelets, and erythrocytes, as well as calcium nanoparticles [58]. They proposed a hypothesis where biofilm formation leads to local injury, followed by inflammation and calcium deposition.

Biofilm formation has also been observed on the surface of salivary stones. In a study on 54 patients with sialolithiasis, biofilm was observed on 71% of the removed stones by fluorescence microscopy, and common oral bacteria were found on half of the stones [60]. The observation that bacterial biofilms were found in 75–100% of patients with clinical post-operative infections, recurrent sialadenitis or pus drainage, indicates that the presence of bacterial biofilms may contribute to more severe cases of sialadenitis.

### 4.2. Recalcitrant Typhoid Fever and Predisposition to Hepatobiliary Cancers

Typhoid fever is an acute food borne illness, predominantly caused by *Salmonella enterica* serovar Typhi that is often characterized by high fever, weakness, headache, abdominal pain, and constipation. Untreated, serious complications may arise, including intestinal bleeding, bowel perforation, septicemia, meningitis, and death [61,62]. There were an estimated 21.7 million cases of typhoid fever worldwide in 2000, resulting in approximately 217,000 deaths [63]. Three to 5% of typhoid fever patients become chronic carriers after the acute phase of the illness [64,65,66]. These chronic carriers are generally asymptomatic and constitute an important reservoir of bacteria that can shed in feces and urine and thereby spread the disease. The chronic carrier state, in which *S*. Typhi is typically detected in the gall bladder, is often associated with pre-existing hepatobiliary disease and approximately 90% of chronic carriers have gall stones [61,64,67,68]. Complications related to chronic carriage of *S*. Typhi include hepatitis, cholecystitis, cholangitis, chronic diarrhea, and pancreatitis as well as hepatobiliary carcinomas [62,69]. Antibiotic treatment generally resolves the acute infection, but it is often ineffective against the chronic colonization of the gall bladder by *S*. Typhi [64,70].

**Figure 1:**
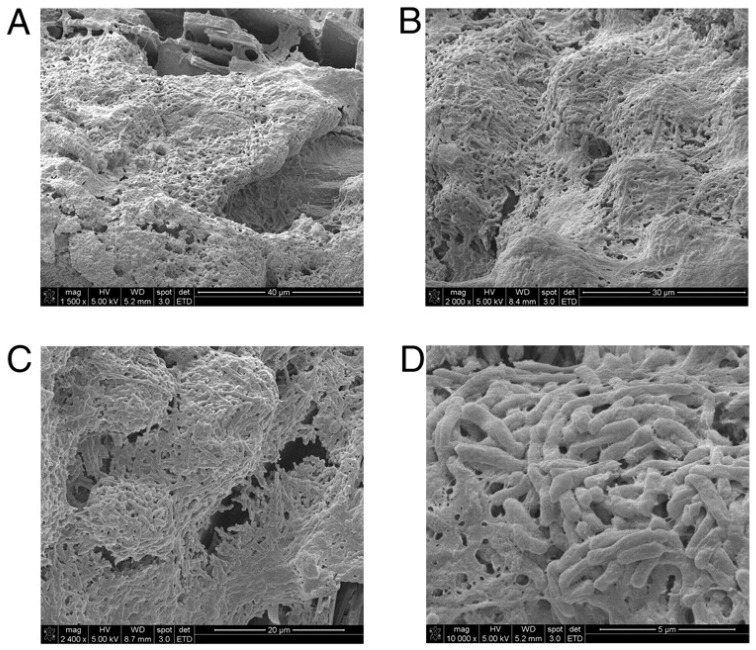
Gallbladder stones from an asymptomatic typhoid carrier in Mexico City support biofilm formation. SEM micrographs show *S*. Typhi embedded in biofilms on the surfaces of gallstones at magnifications of 1500× (**A**), 2000× (**B**), 2400× (**C**), and 16,000× (**D**). R.W. Crawford et al. [73].

There are indications that chronic colonization of the gall bladder by *S*. Typhi, involves attachment and invasion of epithelial cells and biofilm formation on gall stones [48,71,72]. Consistent with this, *S*. Typhi was observed to cover a large part of the surface of gall stones in patients presenting with colilithiasis and asymptomatic typhoid carriage (Figure 1) [73]. This is in agreement with in vitro studies that showed biofilm formation by *S*. Typhi on human gall stones, in a medium supplemented with bile [74,75]. Recent studies indicate that both the host and the bacteria adapt to the chronic gall bladder infection. The host immune response changes from an early pro-inflammatory response to a later anti-inflammatory response [76] whereas *S*. Typhi adapts to the gall bladder environment by increasing the biofilm forming ability and the capacity for persistence and simultaneously reducing the ability to cause acute infections [77,78]. It has also been shown that *S.* Paratyphi A can persist in the gallbladder [79] and accumulating evidence indicate a role for non-typhoidal Salmonellae in persistent infections. Bacteria, other than salmonellae, can occasionally be detected in gallbladder tissues, but gallstones from these patients are generally not covered by bacterial biofilms [73]. This is consistent with the experiments showing that many bacteria causing acute gallbladder infections do not form biofilms on gallstones in the presence of bile [73,80].

Studies performed in a murine gall stone model using *S*. Typhimurium support a role of biofilm formation in the gall bladder in the chronic typhoid carrier state. Mice with diet-induced cholelithiasis had a higher number of *Salmonellae* associated with the gallbladder epithelium and bile compared to gallstone negative controls and this was associated with increased fecal shedding [72,73]. In addition, biofilms covering approximately half the surface of gall stones that were isolated from mice 21 days post infection were detected by scanning electron microscopy. Using the murine gallstones model Gonzales et al. showed that *S*. Typhimurium was more resistant to antibiotic treatment in mice fed a cholelithiasis-inducing diet compared to mice fed a standard diet [81]. These experiments mimicking the chronic carrier state support an important role for biofilms in recalcitrance of chronic *S*. Typhi infections to antibiotic treatment and are consistent with clinical data [70,82,83].

### 4.3. Inflammatory Bowel Disease and Colorectal Cancer

In healthy people, a protective mucosal layer covers the colon epithelium and separates it from the luminal microbiota. Breaches in this protective layer result in increased contact between microbes and the epithelial cells, which can result in biofilm formation on the epithelium. These changes constitute a pathogenic state that has been implicated in development of inflammatory bowel disease (IBD). Studies also suggest an association between colonic biofilm formation, dysbiosis and colorectal carcinogenesis [84,85,86].

IBD (ulcerative colitis and Crohn’s disease) is characterized by chronic inflammation of the digestive tract. Common symptoms are pain, diarrhea, weight loss, and fatigue. Furthermore, patients suffering from IBD have a 10–30% cumulative risk of developing colorectal cancer (CRC) within 30 years after the onset of IBD [87]. IBD has been linked with occurrence of biofilms adhering to the epithelium and dysbiosis of mucosa-associated bacteria that result in stimulation of an inflammatory response. This might be associated with failure of maintaining the integrity of the mucosal barrier resulting in a reduced ability to clear the infection [88]. Using fluorescence in situ hybridization (FISH) and fluorescence microscopy two log higher numbers of bacteria were detected associated with the mucosa of patients with IBD compared to patients with irritable bowel syndrome (IBS; a disease not generally associated with intestinal inflammation) and healthy controls [89]. In addition, the density of bacteria associated with the mucosa has been shown to be significantly higher in patients with intestinal inflammation [88]. Bacteria adhering to colonic epithelium were detected in IBD patients only, and these biofilms were dominated by *Bacteriodes fragilis* [89] and *Enterobacteriaceae* [88]. In contrast, mucosa-associated biofilms with different species composition compared to the IBD associated biofilms were sporadically detected in healthy controls and IBS patients [89]. Several studies have detected higher incidence of *Escherichia coli* in patients with IBD compared to healthy individuals [90] and although biofilms were not demonstrated in vivo, the isolates were isolated from biopsies after removal of the mucosal layer, indicating adherence to the colonic epithelium and the isolates displayed biofilm forming capacity in vitro [90].

Colorectal cancer (CRC) is one of the most common cancer forms worldwide in both males and females [91]. It is generally considered that CRC develops from normal colorectal epithelium as a result of accumulating genetic mutations and epigenetic changes as described by the adenoma-carcinoma sequence model [92]). Accumulating evidence from studies comparing the colonic microbiota compositions of CRC-patients and healthy individuals indicates that CRC is associated with microbial dysbiosis, which is associated with an aberrant inflammatory response ([93,94]).

Based on the adenoma-carcinoma model and the accumulating microbiota data, Tjalsma et al. suggested a bacterial driver-passenger model for CRC [95]. In this model, certain bacteria that are part of the normal microbiota can facilitate DNA damage in epithelial cells that may result in initiation of CRC (bacterial drivers). Biological changes that occur during tumorigenesis result in changes of the microenvironment that favor colonization by opportunistic bacteria (bacterial passengers). The new microenvironment provided by the tumor may result in that the bacterial drivers are eventually outcompeted by bacterial passengers [95]. Recent evidence indicates an important role of biofilms in the colorectal carcinogenesis and Li et al. have suggested that biofilms may be regarded as a driver in the adenoma-carcinoma sequence at an early stage of carcinogenesis [85]. It is currently not known exactly how biofilms influences carcinogenesis, if a specific species is responsible or if it is due to sequential or synergistic activities of different members of the microbial community. However, it has been suggested that accumulation of pro-inflammatory species affects the normal processes of the colorectal epithelium leading to disturbed regulation of inflammation, apoptosis, and cell proliferation. In fact, there are a number of species that have been shown to directly affect these processes. Toxigenic *B. fragilis* has been proposed as an initiator of colorectal cancer due to its ability to disturb the epithelial homeostasis and influence cell proliferation [96,97]. Increased levels of enteropathogenic *E. coli* (EPEC) have been detected in tumors and certain strains have been shown to produce toxins that can induce double strand breaks and cause chromosomal instability [97,98]. Simultaneous colonization of tumor prone mice by toxin-producing *B. fragilis* and *E. coli* resulted in increased DNA damage, faster initiation of tumorigenesis, and greater mortality compared to mice colonized with either species alone, suggesting a potential link between a tumorigenic microbiota and early neoplasia of the colon [97]. *Fusobacterium nucleatum* can influence both inflammatory processes and cell proliferation. It is often found enriched in tumor tissues, and high numbers of *F. nucleatum* correlates with poor prognosis [85,99], however *F. nucleatum* may not be involved in the early stages of carcinogenesis, but may be involved in cancer progression [100,101].

Polymicrobial biofilms adhering directly to the epithelium were detected in colorectal tumors by SEM and FISH [102]. Biofilms were detected in 89% of right-sided tumors compared to 13% of left-sided tumors. Right-sided and left-sided tumors also differ in microbiota composition, molecular characteristics, response to chemotherapy, and prognosis; however it is not clear how these aspects are connected (reviewed by Kim et al. [103]). Tomkovich et al. recently reported that human colon mucosal biofilms were carcinogenic in murine CRC-models, whether the biofilm associated microbiota were from CRC-patients or healthy individuals [104]. Yu et al. detected biofilms associated with CRC, adenomas, and polyps by SEM, FISH, and fluorescence microscopy and found that the prevalence of *F. nucleatum* was significantly higher in biofilms from CRC than the other sample groups [105]. Although a connection between the composition of the colorectal microbiota and CRC is becoming increasingly evident, more research is needed to determine the exact role of the microbial community of the biofilm and its constituting species in carcinogenesis.

## 5. The Integumentary System

### Wound Infections

Wounds are damaging to living tissue caused by e.g., a trauma like cuts, abrasions, burns, and surgery, or as a consequence of underlying illnesses such as diabetes. Most wounds that contain microorganisms heal successfully. However, sometimes microorganisms, and particularly bacteria, multiply, healing is disrupted and wound tissues are damaged resulting in an infection [106,107]. Both chronic and acute wounds are susceptible to infection as a result of the loss of the innate barrier function of the skin and dermal appendages [108]. It is generally accepted that chronic wound infections harbor several different microorganisms and the number of species are thought to be underestimated because of the limitations in culturing techniques. To circumvent this problem, molecular methods can be used to identify viable, but non-culturable bacteria. Also novel microscopy techniques, like e.g., confocal scanning electron microscopy, fluorescence microscopy, and electron microscopy, can be used both in the visualization and to identify bacteria in wounds [108,109]. Chronic wounds can be colonized with several different bacterial species whereas *Staphylococcus aureus* is most commonly isolated [110]. Aerobic bacteria, like *S. aureus*, *S. epidermidis*, and *Pseudomonas aeruginosa*, are often found on the surface of chronic wounds while anaerobic species are predominant in deeper tissue [111]. The anaerobic bacteria that predominate chronic wounds of both humans and animals are *Bacteroides* spp., *Fusobacterium* spp., *Peptostreptococcus* spp., as well as *Clostridium* spp. [108].

As for other bacterial infections, it has historically been assumed that wound infections are caused by planktonic bacteria. More recently, researchers have suggested that chronic wound infections are due to the biofilm mode of growth of the bacteria [2,48]. This idea is supported by recent studies that have demonstrated that chronic wound infections in fact are biofilm infections and observations indicate the presence of biofilms also in acute infections [4,111,112].

It is claimed that bacterial biofilms are located on the surface of wounds and they have been implicated in the failure of wound healing and contribution to chronic inflammation [108]. For instance, it has been suggested that *P. aeruginosa* may significantly contribute to inflammation of the wound by producing rhamnolipids [108]. Biofilm has been postulated to be the reason why wounds like e.g., venous leg ulcers and pressure ulcers often develop into chronic stages [108].

The first scientific study that showed a link between biofilm and wound infections, analyzed the biofilm forming ability of a burn wound isolate of *P. aeruginosa* in vitro using light microscopy [113]. Later, most evidence that wound infections are biofilm related has been shown by different advanced microscopy methods. The study by Bjarnsholt used confocal laser scanning microscopy and a specific peptide nucleic acid- fluorescence in situ hybridization probe to visualize *P. aeruginosa* biofilm in non-healing chronic wounds [4]. The study by Davis et al. used light microscopy, scanning electron microscopy, and epifluorescence microscopy to look for biofilm-like structures in biopsies from wounds on pigs infected with *S. aureus* [112]. A study by James et al. examined biopsies from both acute and chronic wounds using electron microscopy. They found that as many as 60% of chronic wounds contained biofilm, as opposed to only 6% of acute wounds [111]. Lately also an international consensus for clinical indicators of wound infection and biofilm has been published [114]. A total of 14 experts agreed on a list of ten clinical indicators of possible biofilm in a wound, including failure of antibiotic treatment and delayed healing in spite of optimal wound management.

Biofilm infections in wounds are also frequently evaluated macroscopically because of the absence of advanced microscopy equipment and knowledge in clinics. Using macroscopic examination of slough in a wound combined with microscopy of the wound slough has been suggested as a clinical marker of biofilm. Other markers such as a “shiny or sheen” appearance of a wound has also been used as an indication that biofilm is present. These macroscopic observations have the limitation of being highly subjective [108]. A rapid and easy to perform method to diagnose biofilm infections in wounds is needed.

## 6. The Reproductive System

### 6.1. Bacterial Vaginosis

Bacterial vaginosis (BV) is the most common genital tract infection in women during their reproductive years, and tends to have a high rate of relapse and recurrence [115]. Typical for BV are increased numbers of anaerobic bacteria like *Gardnerella vaginalis*, *Atopobium vagnae*, and others accompanied by decreased numbers of protective lactobacilli [116].

Interestingly, since early 1980s one of the criteria used to diagnose BV in clinical practice has been the presence of so called “clue cells”, i.e., epithelial cells covered with bacteria [117]. However, it was not until the studies of Swidsinski et al. thirty years later that the clue cells were finally understood to be desquamated biofilm-coated epithelial cells [118]. In this study, they found that an epithelial, multispesies biofilm dominated by *G. vaginalis* was present in as many as 90% of the BV vaginal biopsies [119]. Currently, it is generally agreed that BV involves the presence of a dense, structured, and polymicrobial biofilm, primarily constituted by *G. vaginalis* clusters strongly adhered to the vaginal epithelium [116]. *G. vaginalis* is probably the first species to adhere to the vaginal epithelium creating a scaffold for other species to adhere [120].

*G. vaginalis* may also be present in the vagina of “healthy” women and sexually inexperienced women and does not necessarily cause BV. Studies by Swidsinski et al. on *G. vaginalis* in urine, showed that the bacteria were attached to desquamated epithelial cells in all patients with proven BV and their partners, and dispersed when it was present in urine of healthy controls [121]. Furthermore, pathogenic strains displayed higher in vitro phenotypic levels of virulence traits like cytotoxicity, adherence, and biofilm production than commensal strains [122]. This might indicate genetic differences between pathogenic and commensal *G. vaginalis* strains. However, recent transcriptome studies have suggested that *G. vaginalis* is able to exhibit different phenotypes through large changes in gene expression [123], and studies on mixed biofilms indicate that expression of *G. vaginalis* virulence genes may be significantly influenced by other bacterial species in the biofilm [124]. Consequently, the composition of the biofilm may also influence the pathogenesis. Accordingly, Verstraelen and Swidsinski suggest that “environmental pressures or ecological disturbances of the vaginal niche might be a more determining factor in biofilm formation and development of bacterial vaginosis in a given woman, than *Gardnerella* genotype alone” [120].

### 6.2. Chronic Endometritis

The uterus has traditionally been assumed to be free of bacteria, but recent studies have identified a functional microbiome of the endometrium under physiological conditions [125,126]. *Lactobacillus* was found to be the most abundant followed by *Gardnerella*, *Prevotella*, *Atopobium*, and *Sneathia*. In approximately 20% of the women investigated, the bacterial community varied greatly from that of the vagina, suggesting that the endometrial and vaginal microbiota are not necessarily identical [127]. In chronic endometritis (CE), the endometrial mucosa may be colonized by common bacteria like *Enterococcus faecalis*, *E. coli*, *G. vaginalis*, *Klebsiella pneumoniae*, *Proteus* spp., *P. aeruginosa*, *Staphylococcus* spp., and *Streptococcus.* spp. [128,129]. The prevalence of CE has been estimated to be 19% in the general population and 45% in infertile patients [125]. However, because CE is often asymptomatic, it is seldom suspected and diagnosed.

**Figure 2:**
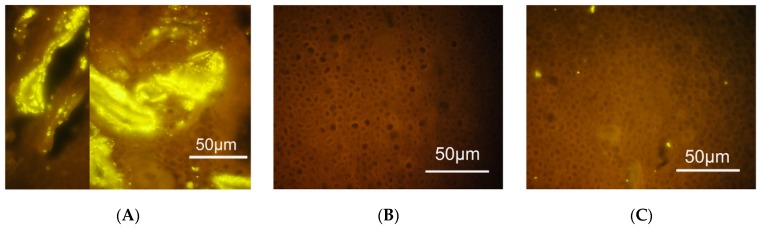
Fluorescence in situ hybridization (FISH) analysis of endometrial samples with probes targeting bacterial vaginosis-associated and other bacteria. (**A**) *Gardnerella* dominated polymicrobial biofilm attached to the endometrium. (**B**) Endometrial sample free of bacteria. (**C**) Bacteria other than *Gardnerella* colonizing the endometrial epithelium. Swidsinski et al. [130].

Swidsinski et al. found that a structured polymicrobial *G. vaginalis* biofilm present in BV often was accompanied by a similar biofilm in the endometrium or the fallopian tubes (Figure 2) [130]. Such biofilms were not found in any of the women without bacterial vaginosis. However, the clinical significance of these endometrial biofilms is still not clear.

Studies performed in horses may shed more light. Bacterial endometritis is an important cause of subfertility in mares, contributing to a major economic loss for the equine industry [131,132]. Ferris et al. used an in vivo model of infectious endometritis where the mares were inoculated with equine *P. aeruginosa* isolates isolated from clinical cases. Bioluminescence imaging of the endometrium displayed focal areas with bacteria surrounded by a “biofilm-like” matrix [133]. Furthermore, the biofilm matrix component Pel and the biofilm-regulatory molecule cyclic di-GMP were detected in such areas. [134]. These observations support the hypothesis of biofilm formation in the uterus by clinical strains.

In addition, Ferris et al. made some interesting observations on the local immune responses in their model. Although inflammatory cells were observed both in areas with and areas without adherent bacteria, neutrophils were decreased and gene expression of the immune-modulatory cytokine interleukin-10 was increased in the areas with biofilm. Whether this modulation of the host immune response is actively caused by the biofilm bacteria, or by the immune system’s reaction to the biofilm structure, is not known.

### 6.3. Mastitis

Mastitis is an inflammatory condition of the mammary gland primarily occurring during lactation. The WHO estimates a global prevalence of approximately 10% of breastfeeding women [135]. *S. aureus* is considered to be the main etiological agent of infectious mastitis, whereas coagulase-negative staphylococci (CNS), *E. coli* and streptococci may also be found. The number of studies dealing with the microbiological aspects of human mastitis is low, especially compared with the vast amount of studies on mastitis in dairy animals. In cattle, mastitis is one of the most frequent and costly diseases in the dairy industry [136]. Also in dairy animals, *S. aureus* is the main pathogen. Other bacteria associated with mastitis are *S. agalactiae* (causing contagious mastitis), CNS, *E. coli*, *Klebsiella* spp., *Enterobacter* spp., *Citrobacter* spp., *S. dysgalactiae*, *S. uberis*, enterococci, and *Pseudomonas* spp. Chronic and recurrent infections are frequent.

The mechanism of persistence of *S. aureus* in its host is still not fully understood. *S. aureus* has been shown to be able to adhere to and internalize into mammary gland epithelial cells in vitro [137]. Intracellular cocci have also been demonstrated in mammary epithelial cells of infected mice, cultured mammary epithelial cells from cows, and epithelial cells isolated from mastitic milk (reviewed by [138]). Invasion may be a way to evade host defenses in vivo. Biofilm formation might be another.

Direct evidence that biofilms are involved in the pathogenesis of mastitis is scarce. However, an in vivo study by Hensen et al. with microscopic examination of *S. aureus* in mammary tissue did indicate the presence of biofilm [139]. Both in the early and chronic stages of infection, clusters of *S. aureus* was observed in the lumen of alveoli or lactiferous ducts, in association with the epithelium. The clusters appeared approximately 24 h after exposure to the pathogen, and polymorphonuclear neutrophils were also often present.

In vitro, both lactose and milk have been shown to increase biofilm formation by *S. aureus* [140,141]. For CNS, a positive correlation between biofilm formation and days in milk was observed, and CNS isolated later in the lactation were better biofilm formers than those isolated earlier [142]. On the other hand, Simojoki et al. found no association between CNS biofilm formation in vitro and the persistence or severity of mastitis in vivo [143].

Bap is a surface protein involved in biofilm formation, and studies on the presence and expression of the *bap* gene may contribute to elucidate a possible role of biofilm in mastitis pathogenesis. The *bap* gene has been identified in up to 25% *S.* aureus isolates and up to 95% of CNS isolates from bovine mastitis [144,145], and up-regulation of the *bap* gene with the presence of low concentrations of milk or lactose in the growth medium has been shown in vitro [141,146]. Zuniga et al. studied the presence of genes encoding Bap and a group of adhesins in staphylococci isolated from subclinical mastitis [147]. The median somatic cell counts, which are markers of sub-clinical mastitis, were higher in milk samples where the bacteria had the *bap* gen and the adhesin genes *eno, fnbA*, *fib*, than in samples with staphylococci without these genes. Thus, the presence of biofilm may contribute to a higher intensity of the inflammatory process. In a study on sub-clinical *S. aureus* mastitis, *bap*-positive isolates were observed to be more able to colonize and persist in the bovine mammary gland in vivo, and anti-Bap antibodies in the serum confirmed that Bap was produced during infection [146] Interestingly, Bap promoted adhesion and prevented entry of *S. aureus* into epithelial cells in vitro, whereas Bap deficient bacteria displayed increased invasion into mammary gland epithelial cells in a lactating mice mastitis model [148]. Altogether, these results may indicate that biofilm formation is correlated with persistence of *S. aureus* in the bovine intramammary gland.

Another indication of the possible contribution of biofilm to the pathogenesis of mastitis is seen in vaccination studies. When mice immunized with formalin-killed biofilm *S. aureus* were compared to those immunized with formalin-killed planktonic bacteria, they showed significantly lower *S. aureus* colonization, as well as less severe clinical symptoms and tissue damage in mammary glands, [149]. Likewise, in a sheep mastitis vaccination study, crude bacterial extracts from strong biofilm formers gave the highest production of antibodies and the best protection against infection and mastitis, when compared with extracts from weak biofilm formers and controls [150].

## 7. The Respiratory System

### 7.1. Chronic Rhinosinusitis (CRS)

Rhinosinusitis (RS) is an inflammation of the nose and the paranasal sinuses, characterized by nasal blockage, obstruction, congestion, or nasal discharge. Additional symptoms may include loss of smell and facial pain and pressure. According to the duration of the disease, it can defined as acute when lasting less than 12 weeks, or chronic when lasting more than 12 weeks [151]. Viruses account for up to 80 to 90% of the acute RS, and the most commonly involved viruses are rhinovirus, respiratory syncytial virus, influenza virus, coronavirus, parainfluenza virus, adenovirus, and enterovirus. The host immune response to a viral infection consists of non-specific and specific components, which will eventually eliminate the invading agent, but also generate dead epithelial and immune cells, creating an environment opportune for secondary bacterial infections. During viral infection in chronic RS, a similar inflammatory process can occur as in acute RS [152,153].

**Figure 3:**
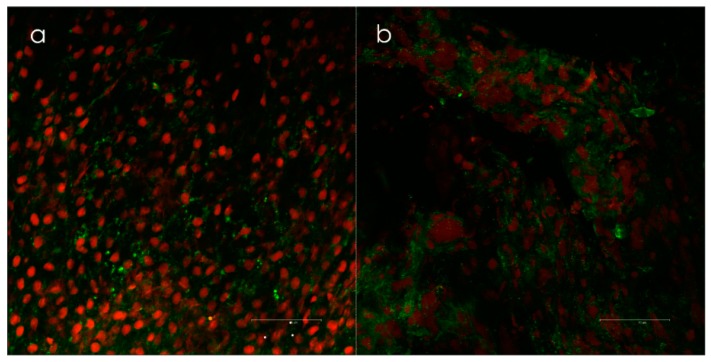
(**a**) Biofilm positive samples taken from the lateral side of concha media visualized by confocal scanning laser microscopy (*Leica TCS SP2 AOBS*) and (**b**) live/dead-staining (Invitrogen’s LIVE/DEAD BacLight™, Invitrogen, Burlington, Canada). Epithelial cells are red, and the bacteria are green. Biofilms were scored when clusters of bacteria with intact membranes were present in both the x-y and x-z axes. Courtesy of Dr. Kjell Arild Danielsen.

The presence of biofilms in CRS patients, as well as in animal models, has been reported in a relatively large number of studies, mainly using scanning electron microscopy and confocal laser microscopy on biopsies (Figure 3) [154,155,156]. Biofilms have also been identified in healthy controls, although to a lesser extent than in CRS patients [157].

Once established, biofilms may induce changes in the mucociliary blanket like destruction of the epithelial layer and absence of cilia, and a local inflammatory response [158,159,160,161]. This suggests epithelial damage being a part of the pathogenesis of biofilm-associated CRS. Interestingly, a study of CRS patients by Tan et al. indicated a link between intracellular *S. aureus* and biofilm colonization [162], indicating that biofilm also can facilitate cellular invasion by pathogens.

The clinical relevance of biofilms in the pathogenesis of CRS has been demonstrated in several studies. The presence of biofilm has been associated with worse pre-operative radiological scores and post-operative outcome, as well as higher risk of recurrence [163,164,165,166]. *S. aureus* biofilm in particular appears to be more pathogenic than other bacterial species, and this has been suggested to be due to a severe local inflammatory response to *S. aureus* superantigens [167].

Colonization in the form of biofilm seems to have a different function in the pathogenesis of *S. pneumoniae* infections. The human nasopharynx is the main reservoir for *S. pneumoniae* and pneumococcal colonization always precedes infection [168]. Striking differences between biofilm residing and dispersed pneumococci indicate that the biofilm phase serves as a non-pathological reservoir. In mouse models, dispersed bacteria displayed inflammatory infiltration, whereas biofilm pneumococci were quickly cleared from the blood without causing invasive disease [169,170]. These observations correspond with gene expression studies where the dispersed cells displayed a higher expression of virulence, stress-response, and bacteriocin production/excretion genes than their biofilm residing counterparts [170].

### 7.2. Pharyngitis and Laryngitis

Pharyngitis, or sore throat, is a very common condition. Most cases are viral, but 10–25% are caused by bacteria. The tonsils and adenoids are lymphoid structures, and recurrent bacterial infections may result in hypertrophy of the tonsillar or adenoid tissue [171]. *S. aureus*, *Haemophilus* spp., and *Streptococcus* spp. are the most common bacterial causes.

Biofilms have been identified in situ after adeno- and tonsillectomy in several studies, using scanning electron microscopy, confocal microscopy, and light and transmission electron microscopy [23,172,173,174,175,176]. Whether biofilm also can contribute to development of clinical symptoms is uncertain, although a couple of studies may indicate an association between the presence of biofilms and chronic inflammation. Al-Mazrou and Al-Khattaf found that biofilms were present in a significantly higher proportion of patients with chronically inflamed tonsils and adenoids than in patients with obstruction [172]. Diaz et al. report that symptoms like harsh raucous sound, tonsillar, and adenoids hypertrophy, apnea, and cervical adenopathies were related to the presence of biofilm in tonsils [174].

Interestingly, studies indicate that adenoid biofilms may also serve as reservoirs for infections in other parts of the respiratory system, as well as in the middle ear. Children with recurrent acute OM were found to have large parts of their adenoid mucosa covered with polymicrobial biofilms containing middle ear pathogens [177]. Similar findings have been reported in children with CRS [178]. On the other hand, in vitro experiments showed that when epithelial cells were covered by *S. oralis* and *S. salivarius* biofilms, the cells were protected from GAS adherence, internalization, and cytotoxic effects [179]. This may indicate protective effects by biofilms produced by such respiratory tract streptococci.

Chronic laryngitis is believed mainly to be non-infectious, and this may be the reason for the sparse research activity on biofilm in relation to this disease. However, when investigating true vocal fold biopsies with confocal scanning laser microscopy and PCR, biofilm was found in 62% of the patients with chronic laryngitis, but only in 20% of the controls, thus supporting a hypothesis that chronic laryngitis also may be biofilm related [180].

### 7.3. Pertussis and other Bordertella Infections

Pertussis, also called whooping cough, is a highly contagious disease, which for decades has been controlled by mass vaccination. Unfortunately, we now observe a resurgence. Pertussis is mostly caused by *Bordetella pertussis*, but also to some extent by human associated *B. parapertussis*. Both are human-specific pathogens, which most likely have evolved from *B. bronchiseptica* or a common ancestor. *B. bronchiseptica*, on the other hand, infects a number of mammal species, including humans, mostly leading to a chronical, subclinical infection. In addition, it causes infectious respiratory disease in dogs and atrophic rhinitis in pigs [181].

*Bordertella* biofilms have been demonstrated in mouse models (reviewed by [182]). In both a nose and a trachea model, distinct architectural features adherent to ciliated epithelium were observed; in the form of mats, towers or pillars for *B. bronchiseptica,* and clusters and macrocolonies for *B. pertussis*. Furthermore, Cattelan et al. observed an association between increased biofilm formation by *B. pertussis* and higher levels of bacterial colonization in the nose and trachea of mice [183]. Direct observations of *B. pertussis* biofilm in humans have not been reported, but abundant extracellular Bordetellae were observed in respiratory tissue samples from 15 infants who had died from confirmed *B. pertussis* pneumonia [184].

Several other data support the notion of biofilm being present in whooping cough. During the biofilm lifestyle, increased production of the surface-associated *B.* intermediate protein A (BipA) can be observed. Expression of the *bipA* gene has also been shown during respiratory tract infection of mice, and anti-BipA antibodies were present in whooping cough patients [185]. When mice were vaccinated with BipA, colonization of the lungs was significantly reduced, and antibodies to BipA were found to opsonize bacteria. Promising results in a murine model have also been obtained with vaccines with other biofilm antigens like BamB and LptD [186]. Consequently, specific biofilm proteins may be candidate antigens for improved pertussis vaccines.

### 7.4. Cystic Fibrosis (CF)

CF was the first infection where biofilm was recognized as part of the etiology, and is probably the most thoroughly studied biofilm infection to date [187]. CF is a genetic disease primarily affecting the respiratory and the digestive system, and is characterized by production of viscid mucus and chronic infections. Lung infection is the main cause of morbidity and mortality [188]. In young patients, primarily *S. aureus* and *H. influenzae* colonize in the airways. *P. aeruginosa* dominates at later stages, although other bacterial species also have been seen to form biofilm in the lungs of CF patients [189].

*H. influenzae* in biofilm-like structures have been observed in lung lavage samples from children with CF [190]. Furthermore, clinical isolates formed biofilms on the apical surface of airway epithelium in vitro, and this stimulated epithelium to increased secretion of factors that mediate inflammation.

Colonization with *P. aeruginosa* often starts with biofilm in the paranasal sinuses, which serves as reservoirs for repeated lung infections that finally become chronic. *P. aeruginosa* biofilm has been observed in lung tissue, lung abscess, and sputum of CF patients [191,192,193,194]. Microscopic analyses have shown that *P. aeruginosa* in sputum grows as microcolonies adherent to sputum components [195]. In response to the presence of biofilm, large numbers of polymorphonuclear leukocytes (PMNs) infiltrate the area, producing a chronic inflammation with subsequent tissue damage, loss of lung function, and obstruction of the airways. The metabolic activity of bacteria and cells consume available oxygen and produce anerobic conditions [195], which unfortunately seems to favor the biofilm mode of *P. aeruginosa* even more [196].

**Figure 4:**
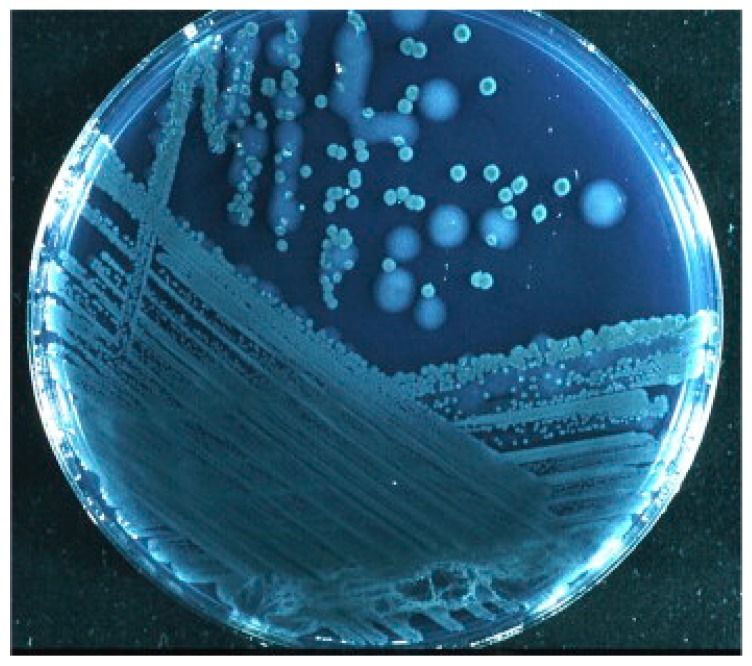
*P. aeruginosa* from sputum of a cystic fibrosis patient. Mucoid (large) and nonmucoid (small) colonies. The mucoid variant over-produces alginate, which is the matrix in the *P. aeruginosa* biofilm in the respiratory tract of cystic fibrosis patients. Mucoid colonies are only found in patients with chronic biofilm infection and alginate from mucoid colonies is therefore a biofilm-specific antigen. Høiby et al. [197].

Diagnostic criteria for *P. aeruginosa* biofilm infection in CF patients are recommended in the ESCMID guideline for the diagnosis and treatment of biofilm infections of 2014 [197]. One criterion is the detection of bacterial aggregates embedded in an alginate-including matrix in sputum or other samples from the lower airways. Mucoid growth with hyperproduction of alginate by bacteria isolated from lung tissue and sputum is also considered diagnostic for biofilm infection (Figure 4). The presence of alginate in sputum is a good indication. Serum IgG antibodies to *P. aeruginosa* antigens, including the major biofilm matrix component alginate, is usually present. Likewise, sIgA in saliva or in the mucosa of the paranasal sinuses indicates biofilm in this location.

## 8. The Urinary System

### 8.1. Chronic Bacterial Prostatitis

Bacterial prostatitis (BP) generally presents with urinary tract infection (UTI), pain in the pelvic and genital region, and occurrence of bacteria in expressed prostatic secretions. Acute BP may result in complications such as reduced fertility, bladder infections, prostatic abscesses, urosepsis, and death. The disease can also progress to chronic prostatitis (CP) or chronic pelvic pain syndrome (CPPS) [198,199]. If symptoms of bacterial prostatitis last for more than 3 months, it is considered to be chronic bacterial prostatitis (CBP). Similar to UTI, the species most commonly associated with BP are *E. coli*, *Proteus mirabilis*, *P. aeruginosa*, *Klebsiella* spp. and other Enterobacteriaceae as well as *E. faecalis* [199,200,201]. Pathogenesis of CBP has been suggested to involve biofilm-forming bacteria [202,203,204,205]. This hypothesis is based on the observations that bacteria can persist in the prostate for long periods of time, a high percentage of cases are refractory to treatment with antibiotics [200,202], and even when the treatment is successful, as judged by negative microbiological tests, symptoms may remain [202]. A few studies support the involvement of biofilm forming bacteria in the pathogenesis of CBP. Nickel et al. used an animal model of experimental BP and showed that chronic symptoms correlated with the presence of glycocalyx-enclosed bacterial microcolonies inside the ducts and acini of the prostate [206]. Occurrence of microcolonies seemed to be associated with a chronic inflammation of the gland. In support of this finding; scanning electron microscopy of prostate calcifications from patients with CBP showed structures reminiscent of bacterial microcolonies on the surface of, and embedded in, the calcifications [205,207]. These observations were associated with isolation of bacteria, from the surface of calcifications, with the potential to form biofilms in vitro [207]. In addition, prostatitis appears to be correlated with occurrence in urine of *E. coli* with high capacity to form biofilm in vitro [203,204]. However, biofilm formation may not be the only explanation for persistent bacterial infections in the prostate. Uropathogenic *E. coli* (UPEC) strains, which are often isolated from BP-patients, can invade prostate cells in a mouse model (NOD and C57BL/6J mice) as well as in cultured RWPE-1 and PEC-1 cell lines [208,209]. In the mouse model, the UPEC strains were able to proliferate within the epithelial cells following invasion, and although the effect was strain dependent, the infection could induce and sustain pelvic pain in the NOD mice [209]. UPEC strains can also invade bladder epithelial cells, proliferate and form matrix-enclosed biofilm-like structures that has been referred to as intracellular bacterial communities (IBC) [210,211]. Interestingly, the invasive capacity of different UPEC strains into the prostate cell line RWPE-1 correlated with both the ability to adhere to epithelial cells and the capacity to form biofilm on a plastic surface, in vitro [208] and similar results were observed with an adherent/invasive *E. coli* (AIEC) strain, which is highly similar to UPEC, isolated from an ileal lesion in a Crohn’s disease patient [212]. It is therefore possible, but to our knowledge has not been demonstrated, that bacteria can persist in the prostate using a similar strategy. More research is needed to understand the pathogenesis of chronic bacterial prostatitis and if bacterial persistence involves biofilm formation.

### 8.2. Urinary Tract Infections (UTI)

UTIs are very common infections in humans and occur when bacteria, often from the rectum and perineum, enter the urethra and colonize the urethra, bladder, ureters, and/or kidneys. Symptoms depend on the anatomical location of the infection and generally include urinary frequency, urgency, dysuria, and/or pain in the lower abdominal region. Systemic symptoms and sepsis may occur, especially in infections involving the kidneys. A urinary tract infection often clears up on its own within a few days or after a short course of antibiotic treatment, but the relapse rate is high. The etiology of these chronic, or recurrent, urinary tract infections is not fully understood and has been attributed to reintroduction of bacteria via the rectal-peritoneal route, or from a vaginal reservoir. However, a growing body of evidence suggests involvement of a persistent infection in the bladder due to IBC [213,214,215,216,217,218,219].

IBC were first described in a mouse model using an immunosuppressed mouse strain and UPEC [210], the primary UTI-pathogen accounting for 70–95% of cases. Scanning electron microscopy, transmission electron microscopy, and confocal laser scanning microscopy combined with fluorescently labeled bacteria and specific staining methods for type I fimbriae and polysaccharides revealed large protrusions on the urothelium which consisted of bacterial populations surrounded by a matrix of polysaccharide and fimbrial proteins that were not localized to specific organelles [210]. These results indicate bacterial biofilm formation inside the bladder epithelial cells. IBC can facilitate bacterial immune evasion as shown by video microscopy where luminal bacteria of the bladder were consumed by neutrophils whereas bacteria that had invaded the epithelial cells were protected from neutrophil attack and could proliferate in the IBC [220]. In the mouse model, UPEC undergo three-stage cycles of invasion of epithelial cells, followed by proliferation as IBC and dispersion into the bladder lumen, which allows for a new round of infection of adjacent cells [220]. This mechanism could explain persistent UTI with periods of quiescent and relapsing infections.

**Figure 5:**
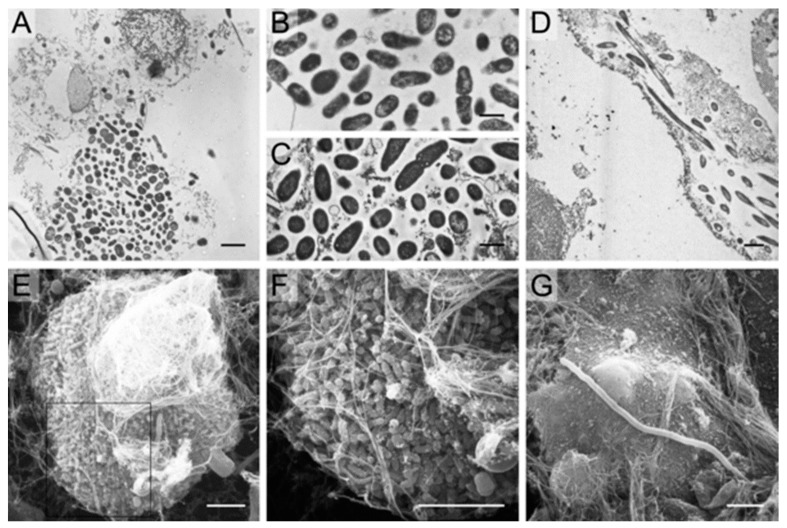
Electron microscopy findings in urines from women with cystitis. TEM analysis of human cystitis urine specimens (**A**) revealed large collections of bacteria associated with nuclei and other cellular debris. These collections of bacteria from human urines (**B**) have similar morphology and organization as those recovered from intact murine intracellular bacterial communities (**C**). Bacteria and filaments were also observed intracellularly within exfoliated epithelial cells in a urine sample quickly fixed and analyzed from an *E. coli* cystitis patient (**D**). SEM analysis of cystitis urines deemed positive for IBCs and filaments captured large bacterial biofilm-like collections (**E**,**F**) composed of bacteria with a smaller, more coccoid morphology than typical *E. coli*. Long filaments were also captured by SEM (**G**). Scale bars, 2 μm (**A**,**D**), 1 μm (**B**,**C**), and 5 μm (**E**–**G**). Rosen et al. [222].

There is also evidence that IBC can contribute to chronic and recurrent UTI in humans [213,217,221,222,223,224], and IBC has been detected in the epithelium of the urinary bladder. The majority of UPEC isolated from urine of women with urinary tract infections were able to form IBC in a mouse model [213,221]. In addition, IBC have been observed by electron microscopy in shed epithelial cells in urine from 18% of women presenting with acute UTI (Figure 5) [222]. In this study, IBC were not detected in the urine samples from asymptomatic women. IBC have also been detected in 36.8% of urine samples from children presenting with cystitis [223] as well as in 44% of renal transplant patients tested for UTI [224].

## 9. Conclusions

**Figure 6:**
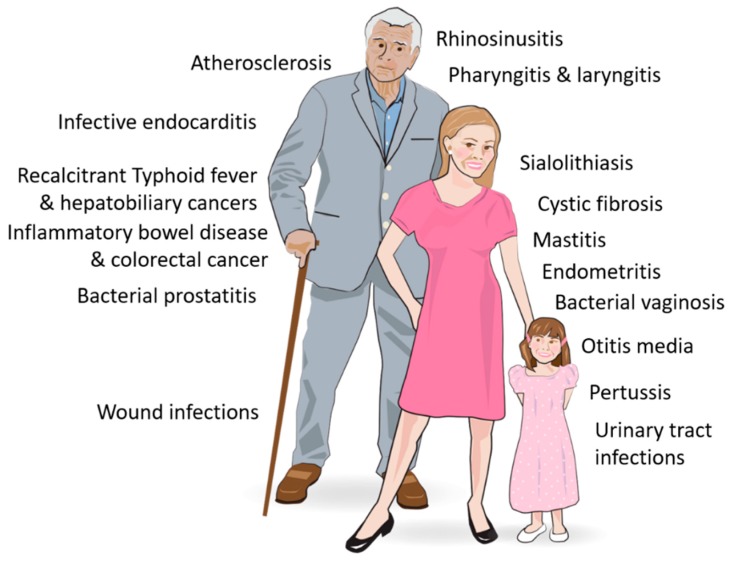
Diseases associated with bacterial biofilms.

An increasing number of diseases have been suggested to be biofilm related. In the majority of cases, this is based on observations of biofilm-like structures in biopsies, autopsies, and exudates of patients and /or research animals (Figure 6). Additionally or alternatively, the presence of biofilm is indicated by studies on bacterial phenotypes during infection, immune responses, and vaccination experiments. However, even when biofilm is present, there is still a question whether the biofilm is the cause of the disease or the bacteria are just taking advantage of a favorable environment for colonization caused by the disease.

Our review of the literature shows that biofilm may potentially contribute to the pathogenesis of a disease in several ways. Biofilm formation increases the bacteria’s resistance against the defense mechanisms of the body, as well as antimicrobial treatments, thereby promoting chronic infections. Biofilms may also function as an environment that accumulate different bacterial species as well as bacterial numbers in certain locations. This can result in deleterious effects on host cells due to concentrated, sequential, and/or synergistic activities by the present bacteria. Furthermore, the mere presence of persistent biofilms may modulate the local immune response in several ways, e.g., by stimulating a local inflammatory response that can cause or aggravate tissue damage. These biofilm-mediated mechanisms have also been suggested to be involved in initiation and/or progression of cancers, such as CRC. In wounds, an additional effect of biofilm is that the physical presence may obstruct wound healing. Biofilm might also facilitate cellular invasion by pathogens, as indicated by the observed link between intracellular *S. aureus* and biofilm colonization in the upper airways. Interestingly, biofilm-like intracellular bacterial communities have been identified, e.g., within the epithelial cells of the bladder of the urinary tract, which appears to result in bacterial immune evasion and persistent or recurrent infections.

Presence of biofilm can be linked to the severity and prognosis of disease, e.g., as in chronic rhinosinusitis and cystic fibrosis. However, colonization in the form of biofilm may also serve as a sub-clinical reservoir for pathogens preceding clinical infection with planktonic bacteria, as observed for *S. pneumoniae* in the nasopharynx and UPEC in the urinary bladder. In addition, biofilms by non-pathogenic bacteria may even offer protection against pathogen infection, as observed for biofilms by *S. oralis* and *S. salivarius* in the upper airways.

Based on present research, it is clear that both diagnosis and treatment of a number of chronic diseases need to take into account the importance of biofilm. Diagnostic criteria for biofilm infections are needed, and have already been suggested for a few diseases like cystic fibrosis and chronic wounds. Development of effective treatment against such infections is also imperative.

## Figures and Tables

**Table 1:** Biofilm-associated diseases of different body systems and their affected organs.

Body System	Affected Organs	Disease
Auditory	Middle ear	Otitis media
Cardiovascular	Cardiac valves	Infective endocarditis
	Arteries	Atherosclerosis
Digestive	Salivary glands	Sialolithiasis (salivary duct stones)
	Gall bladder	Recalcitrant typhoid fever and predisposition to hepatobiliary cancers
	Gastrointestinal tract, especially the small and large intestine	Inflammatory bowel disease and colorectal cancer
Integumentary	Skin and underlying tissue	Wound infections
Reproductive	Vagina	Bacterial vaginosis
	Uterus and fallopian tubes	Chronic endometritis
	Mammary glands (breasts)	Mastitis
Respiratory	Nasal cavity and paranasal sinuses	Chronic rhinosinusitis
	Throat, i.e., pharynx with tonsils and adenoids, and larynx with vocal cords	Pharyngitis and laryngitis
	Upper and lower airways	Pertussis (whooping cough) and other Bordertella infections
	Upper and lower airways	Cystic fibrosis
Urinary	Prostate gland	Chronic bacterial prostatitis
	Urethra, bladder, urethers, kidneys	Urinary tract infections
